# Acute Respiratory Distress Syndrome after Treatment of Metastatic Prostate Cancer with Taxotere: A Case Report and Literature Review

**DOI:** 10.1155/2015/198381

**Published:** 2015-08-13

**Authors:** Ali Raufi, Jennifer Dotson, Mohamad Khasawneh

**Affiliations:** Division of Hematology-Oncology, Department of Medicine, Joan C. Edwards School of Medicine, Marshall University, Edward Comprehensive Care Center, Huntington, WV 25701, USA

## Abstract

Prostate cancer is the most common cancer in men. Docetaxel is a common chemotherapeutic agent that has proven its efficacy in the treatment of patients with both castration sensitive and resistant metastatic prostate cancer. We report a case of acute respiratory distress syndrome (ARDS) in a patient with metastatic prostate cancer treated with docetaxel (Taxotere). ARDS is very rare but life threatening complication of docetaxel which requires aggressive supportive care and close monitoring. Better awareness and prompt diagnosis of this treatment related ARDS will improve the effectiveness and outcome of its management.

## 1. Introduction

We present a case of acute respiratory distress syndrome in a patient with metastatic prostate cancer treated with docetaxel (Taxotere). Docetaxel is a common chemotherapeutic agent used in the treatment of patients with castration resistant and sensitive metastatic prostate cancer. Docetaxel has many adverse reactions but acute respiratory distress syndrome (ARDS) is relatively rare and life threatening complication. There are very few case reports of ARDS after administration of docetaxel; however in those cases, docetaxel was given in combination with gemcitabine, an agent that has also been reported to cause ARDS in the past.

## 2. Case Presentation

We report a 74-year-old white male who was diagnosed with de novo metastatic prostate cancer. He was started on concurrent hormonal therapy (Lupron) with docetaxel (based on the ECOG 3805 trial). The dose of docetaxel was reduced due to neuropathy. After cycle # 3 of chemotherapy, he presented to the emergency room with fever, fatigue, and generalized weakness. He had a chest X-ray that showed “bilateral lower lobe atelectasis versus infiltrate.” Labs showed absolute neutrophil count of 200 cells/*μ*L and platelets 67,000 cells/*μ*L. Treatment was initiated with empiric cefepime and vancomycin.

**Figure 1:**
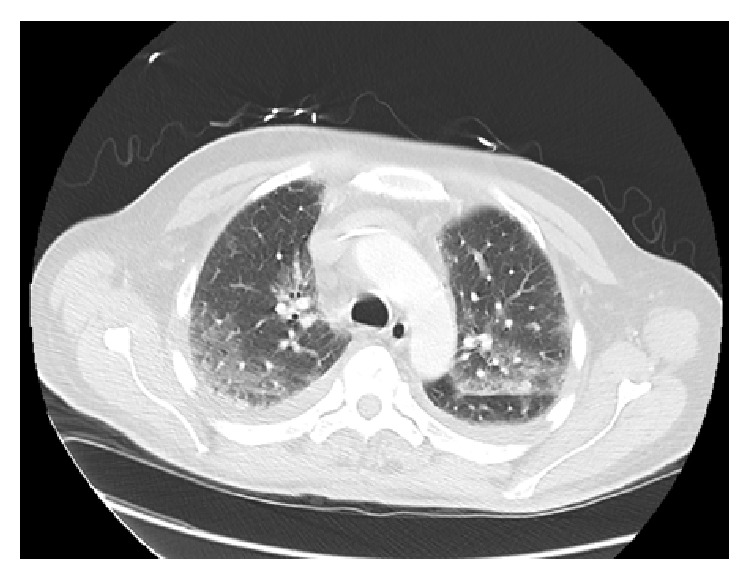
Diffuse ground glass opacities, pulmonary edema. There are small bilateral pleural effusions.

CT of the chest in Figure 1 was done for further evaluation and was consistent with pulmonary edema. There was no infiltrate to suggest acute pneumonia. He had no history of cardiac disease or congestive heart failure prior to starting chemotherapy. He became hypoxic on the third day of admission. Echo was obtained that showed preserved ejection fraction. Diuresis was initiated with Lasix but patient's respiratory status did not improve. Blood, urine, and sputum cultures were all negative and no other source of infection was found. He was subsequently transferred to the ICU and required intubation for persistent hypoxemia. Chest X-ray showed worsening diffuse bilateral edema. PaO_2_/FIO_2_ ratio was >200 mmHg suggestive of acute respiratory distress syndrome. The patient was started on high dose steroid and continued on supportive management. He became more awake and alert. The patient was eventually extubated and started on Continuous Positive Airway Pressure (CPAP). Weaning was unsuccessful and he needed continuous CPAP. The patient did not want further aggressive management and requested palliative care. He eventually enrolled in hospice program.

## 3. Discussion

Prostate cancer, the most common cancer in men, accounts for 220,800 cases and approximately 27,540 expected deaths per year in the United States for 2015 [1]. The spectrum of advanced systemic disease ranges from pathologically identified locoregional nodal metastases at the time of radical prostatectomy to widespread systemic involvement. The bone, pelvis, and abdominal lymph nodes are the most common sites of metastatic disease. The five-year overall survival in metastatic prostate cancer is 28 percent [1].

Androgen deprivation therapy (ADT) is historically a standard part of the initial approach for patients with metastatic prostate cancer. On the other hand chemotherapy like docetaxel has been reserved for patients who have progressed on ADT [2]. The SWOG 9916 trial [3] and TAX327 trial [4] were the pioneer in showing a survival advantage with chemotherapy in metastatic prostate cancer favoring docetaxel group. The results of two large randomized trials have changed the role of chemotherapy considerably in 2004 in patients with castration resistant metastatic prostate cancer treated with docetaxel-based chemotherapy by demonstrating an overall improvement in survival.

Investigators from the SWOG 9916 trial randomized patients to receive mitoxantrone plus prednisone versus docetaxel (60 mg/m^2^) plus estramustine (Emcyt) and dexamethasone every 3 weeks. This trial demonstrated a survival benefit of 2 to 3 months in the docetaxel arm.

Tannock et al. showed a similar survival benefit of 3 months in another international randomized phase III trial (TAX327). The patients were randomized to receive docetaxel (at 75 mg/m^2^) plus prednisone given every 3 weeks compared with mitoxantrone and prednisone. These trials led to various studies in which the role of docetaxel was evaluated in combination with newer agents [5–7]. A recent early analysis of ECOG-led phase III randomized trial has shown improved survival in men with castration sensitive metastatic prostate cancer (*n* > 900) and a high metastatic burden (visceral disease, high bone burden) who received six cycles of docetaxel given at the start of standard ADT compared with patients who received ADT alone. The upfront combination chemohormonal therapy produced a statistically and clinically significant improvement in overall survival compared with ADT alone (52.7 versus 42.3 months with *p* value 0.0006, Hazard ratio 0.63 (0.48, 0.82)) [8]. The median OS had not been reached in men with low-volume disease at the time of the analysis.

The taxanes, paclitaxel (Taxol), and docetaxel (Taxotere) have demonstrated their effectiveness as first-line treatment setting for metastatic breast, lung, ovarian, and GI cancers in many randomized clinical trials. They have emerged as important and widely prescribed chemotherapeutic agents exhibiting broad range of antitumor activities [9–13].

Paclitaxel was isolated in 1971 from the Pacific yew (*Taxus brevifolia*) whereas docetaxel, a second-generation semisynthetic taxane analogue from the European yew (*Taxus baccata*), was identified in the 1980s. These antimicrotubulin drugs act mostly in M phase of the cell cycle. They interfere with the normal function of microtubule growth, by hyperstabilizing their structure resulting in inhibition of DNA, RNA, and protein synthesis [14].

The lung is a frequent target and a common form of iatrogenic injury due to antineoplastic agents [15, 16]. Antineoplastic related lung toxicity has a wide spectrum that varies from interstitial pneumonitis, organizing pneumonia, radiation recall pneumonitis, and nonthromboembolic pulmonary hypertension to diffuse alveolar damage, alveolar hemorrhage, and noncardiogenic pulmonary edema [17–22]. The common patterns of taxane related lung toxicity vary from diffuse interstitial pneumonitis to relatively rare capillary leakage that could be life threatening. Docetaxel has been associated with fluid retention manifested as peripheral edema, pleural effusion, and ascites [23, 24]. The fluid retention is a cumulative-dose process that is delayed by administration of corticosteroids [25]. Noncardiogenic pulmonary edema has been reported with docetaxel [26, 27]; however in both cases docetaxel was given in combination with gemcitabine, an agent that has also been reported to cause ARDS in the past [28]. It was not clear in the above case reports if ARDS was associated with a single agent or was the consequence of a synergy between the two chemotherapeutic drugs. Above, we have described a case of a patient on docetaxel who experienced ARDS without any other discernible causes. Infection had been effectively ruled out and there was no evidence of trauma or heart failure. Respiratory symptoms started within one week of chemotherapy. Our patient received docetaxel as sole chemotherapeutic agent [26, 27]. Gonadotropin-releasing hormone (GnRH) agonist (Lupron) was continued in our patient as hormonal therapy along with the docetaxel. Hormone therapy usually does not cause ARDS; however there is one case report published in 1995 in which patient developed ARDS after injection of leuprorelin [29]. It is unclear if the combination of hormonal therapy with docetaxel contributed to the development of ARDS.

The management of drug induced ARDS required early recognition of pulmonary toxicity and discontinuation of chemotherapy like taxane along with prompt initiation of high dose corticosteroid and diuretic and supportive care. These patients need aggressive supportive care and close monitoring [30]. Bringing awareness of cases such as these may help haste the diagnosis of other patients presenting with similar signs and symptoms, therefore aiding in a quicker recovery.
